# When the Media Omits or Includes Scientific Evidence in Its Publications: Science and Battles on X about Child Sexual Abuse

**DOI:** 10.3390/children10121857

**Published:** 2023-11-26

**Authors:** Ane Olabarria, Ana Burgués-Freitas, Ane López de Aguileta, Harkaitz Zubiri-Esnaola, Elisabeth Torras-Gómez, Mar Joanpere, Garazi López de Aguileta, Garazi Álvarez-Guerrero, Emilia Aiello, Cristina Pulido, Gisela Redondo-Sama

**Affiliations:** 1 Department of Didactics of Language and Literature, University of the Basque Country, 20018 San Sebastian, Spain; ane.olabarria@ehu.eus (A.O.); harkaitz.zubiri@ehu.eus (H.Z.-E.); 2 Department of Sociology, University of Granada, 18071 Granada, Spain; anaburgues@ugr.es; 3 Social Work Training and Research Section, University of Barcelona, 08034 Barcelona, Spain; alopezdeaguileta@ub.edu; 4 Vicerectorat de Recerca, Universitat de Barcelona, 08007 Barcelona, Spain; etorras@ub.edu; 5 Department of Business Management, University Rovira i Virgili, 43204 Reus, Spain; mar.joanpere@urv.cat; 6 Department of Curriculum & Instruction, University of Wisconsin-Madison, Madison, WI 53706, USA; lopezdeaguil@wisc.edu; 7 Faculty of Education and Sport, University of Deusto, 48007 Bilbao, Spain; garazialvarez@deusto.es; 8 Department of Developmental and Educational Psychology, University of the Basque Country, 20018 San Sebastian, Spain; 9 Department of Sociology, Autonomous University of Madrid, 28049 Madrid, Spain; emilia.aiello@uam.es; 10 Department of Journalism and Communication Sciences, Autonomous University of Barcelona, 08193 Barcelona, Spain; cristina.pulido@uab.cat; 11 Department of Pedagogy, University Rovira i Virgili, 43007 Tarragona, Spain

**Keywords:** child sexual abuse, media information, scientific evidence, social impact

## Abstract

Scientific evidence of social impact demonstrates how violence against children is successfully prevented. Currently, the scientific research on social impact has a focus on the analysis of actions that succeed in the implementation of such scientific evidence. This article is based on scientific research that looks at which media actions help or hinder the implementation of evidence-based actions to solve the most sensitive social problems. The social media analytics methodology has identified the posts and reposts generated during two consecutive days by news articles published by three newspapers about the official report on child sexual abuse in Spain. Their analyses have been made through communicative methodology, including voices of adult victims or survivors of child sexual abuse. The results indicate that media information that omits scientific evidence of social impact provokes battles between diverse ideological groups, while information based on scientific evidence of social impact generates consensus among people from different ideologies and actions oriented to overcome the problem.

## 1. Introduction

Violence against children (hereafter, VAC) is an increasing concern in today’s societies, often being the topic of news articles, social media debates, and daily conversations. VAC involves physical, sexual, and emotional abuse and neglect by parents and other adults towards anyone under 18 years of age. Violence between peers, intimate partner violence, bullying, and violence related to teachers, both in person and online, are also included in this definition.

There is a whole body of literature on the breadth and depth of VAC, showing how many lives it impacts: every year, more than 1 billion children in the world suffer violence [1]. According to the WHO’s cross-national Health Behaviour in School-aged Children (HBSC) study, involving 45 countries and regions in Europe and Canada in 2017/2018, one in ten teenagers experienced victimization at least once during the previous two months [2].

A form of VAC that has been widely studied is bullying [3,4]. The 2018 PISA results [5] show that 23% of students in OECD countries acknowledge having been victims of physical, verbal, or relational bullying at least a few times a month. This same report shows that male students and those with lower reading performance were more likely to report experiencing bullying than their female counterparts and high-achieving students.

Another form of VAC which has prompted considerable attention within the scientific community is that of child sexual abuse (CSA). The numerous studies available show that CSA is present in diverse settings, including homes [6,7], schools [8,9], religious institutions [10], and broader communities [11]. The most prevalent context for children to encounter physical and emotional violence is typically within their own homes [12,13]. In this vein, a study analyzing testimonies of 870 survivors of CSA within families in Germany (IICSA) underscores the shared social rules within families that allow abuse to persist.

Research has also shown the prevalence of CSA in broader communities. This includes, among other contexts, sports. It is estimated that the prevalence of survivors of CSA is between 2% and 10% [14]. Some studies have pointed out the trustful relationships between coaches and athletes, as well as the physical contact involved in some sports modalities, as risk factors [15]. However, coaches are not the only perpetrators. A literature review on seven studies conducted in six different countries found that, although the coach was the perpetrator in most cases, some victims also reported sexual abuse by fellow athletes, medical staff, board members, and other staff involved in sports [16].

Another context in which the prevalence of CSA has been studied is within religious institutions. A study [13] carried out in France focusing on CSA related to the Roman Catholic Church found this to be the second sphere of perpetration in terms of prevalence in the French context. However, such prevalence has significantly dropped since the 70s, in line with a Dutch case, where the current estimated prevalence of this phenomenon was only a fraction of the prevalence rate of non-familial CSA [17,18]. In all cases, unlike what happens in other spheres, men were more affected than women [13,17,18].

Along with the prevalence of VAC, the consequences of suffering violence or being exposed to it in childhood have also been widely studied. VAC can have a negative impact on children’s normal development, leading to both harmful physical and mental consequences. Some of the consequences it generates include experiencing academic difficulties, including problems with retention [19,20,21], and being more vulnerable when developing physical and mental health [22,23]. This can lead to self-harm and even suicidal ideation [24,25]. Furthermore, it has been shown that these consequences can persist over time [26]. In the particular case of CSA, an umbrella review [27] investigating the association between childhood sexual abuse and various long-term psychiatric, psychosocial, and physical health outcomes revealed that childhood sexual abuse is significantly associated with outcomes including various psychiatric diagnoses, negative psychosocial outcomes, and physical health conditions. The analysis encompassed 559 primary studies and 28 outcomes involving 4,089,547 participants. Therefore, addressing, preventing, and overcoming CSA from an early age is essential to provide children with opportunities to have a healthy and happy future [20,28,29].

### 1.1. Scientific Evidence of the Social Impact of Violence against Children

Despite the devastating implications of VAC, there is much that schools and other contexts where children interact can do to prevent and overcome it. Scientific evidence of social impact has already shown some actions that are currently making this a reality [30,31,32,33]. Scientific evidence of social impact refers to the evidence published in indexed scientific journals showing progress towards the achievement of democratically set goals, such as sustainable development goals [31,34,35]. In the particular case of VAC, scientific evidence of social impact includes the actions that contribute to achieving the goal of good health and well-being by preventing and diminishing the effects of such violence. 

In light of the relevance of overcoming VAC, the European Commission prompted the elaboration of the report “Achieving student well-being for all: educational contexts free of violence” [31]. Beyond an analysis of the negative consequences of all types of VAC, the report presents the actions and programs aimed at tackling VAC for which there exists scientific evidence of success. According to the analysis, such interventions share some common characteristics. These include being based on scientific evidence of social impact, involving and training the whole educational community, promoting friendships and bystander intervention, having zero tolerance for all types of violence (including CSA), and building resilience, including beyond formal educational settings in emergency scenarios, such as the COVID-19 pandemic.

Along these lines, the scientific literature available shows that one of the actions to have yielded successful results in overcoming VAC in diverse contexts is the Dialogic Model of Prevention and Resolution of Conflicts [30,36,37,38]. Within this model, which involves rejecting violent behaviors through an egalitarian dialogue, the literature has shown the social impact of the Zero Violence Brave’s Club [32,39,40]. One of the key aspects of the Dialogic Model and particularly of the Zero Violence Brave’s Club is attributing attractiveness to speaking up, reporting situations of violence, and standing by the side of victims [32]. In addition, the whole educational community is involved and trained through the scientific evidence of social impact, both inside and outside of schools [36]. Successfully implementing this action has shown to increase the number of reported instances of violent situations in many schools [38], promote the creation of friendship networks [32], and reduce violence overall in the studied schools where they have been applied [28,30,41]. Furthermore, due to its social impact, the Zero Violence Brave’s Club has been included in the European Toolkit for Schools [39]. 

The founding members of the Zero Violence Brave’s Club are students who do not develop hierarchical leadership but, on the contrary, dialogic leadership [42], giving protagonism to all their classmates. Doing so, the Zero Violence Brave’s Club is increasing the number and diversity of its members until it is no longer necessary because the whole community develops this activity. The same dialogic leadership has been identified in social networks promoted using scientific evidence of social impact. For instance, during the first year of the pandemic, it was identified that on Twitter there were more fake instances of scientific evidence of COVID-19, but those based on scientific evidence had many more reposts than the ones based on false information [43].

The scientific literature has also shown the social impact of the “open doors” action. This has shown to foster safety, improve communication channels with diverse adults and other peers, and provide strong coordination between the school and the whole community with the goal of overcoming all types of VAC [33]. The social impact of teacher training about VAC based on scientific evidence of social impact has also been published, showing an increase among teachers of awareness of this violence and preparedness to intervene in educational settings in similar situations [41,44]. In addition, dialogic feminist gatherings, in which students read and engage in egalitarian dialogues around scientific evidence of social impact, have been demonstrated to contribute to overcoming gender-based violence among underage girls [45,46,47]. Nevertheless, despite the wealth of scientific literature of the actions that are successfully preventing and overcoming VAC in diverse contexts in different countries, there are still hindrances to implementing them in many other contexts.

### 1.2. The Impact of Journalism

Journalism can either contribute to or hinder social change [48,49,50,51]. So-called “yellow”, “sensationalist”, or “tabloid” journalism is often driven by ideological perspectives and interests unrelated to the overcoming of social issues. In this sense, research found that, for instance, in the case of Ukrainian “high-quality media”, journalists frequently did not consider if topics launched had the purpose of manipulating or agenda-setting, if the published information was newsworthy [52]. Such behavior can not only influence agenda-setting, as the authors mention, but can generate controversies and debates that impede the dissemination and implementation of scientific evidence of social impact, which is crucial for overcoming sexual abuse [53]. There are even instances of “yellow journalism” attacking victims of sexual violence [54] or negatively framing religious groups, negatively influencing some sectors of the population [55,56].

Moreover, journalism engaging in such sensationalist practices has consequences on user behavior on social media. For instance, a study found that tabloids played a role in fueling democratically dysfunctional misinformation and disinformation behavior among users sharing these kinds of news on social media [57]. 

In contrast, a significant portion of journalism focuses on presenting facts and protecting victims [58,59,60], and this makes it easier for journalism to have a positive effect on the public [61]. In particular, when the media presents scientific evidence of social impact, it is easier for people from diverse backgrounds to focus and agree on effective actions and their implementation [53]. Furthermore, journalism with such a social impact aligned with the sustainable development goals plays a significant role in rebuilding trust in the field of journalism [62,63].

However, within the framework of VAC, and more specifically, in the context of CSA, the influence of journalism on promoting or impeding prevention and recovery efforts remains underexplored. This article aims to address this gap by analyzing the media actions that either facilitate the application of scientific evidence of social impact in preventing and addressing VAC or, conversely, hinder such progress. 

## 2. Materials and Methods

The objective of this qualitative study is to explore which media actions facilitate or deter the implementation of evidence-based solutions to social problems of utmost sensitivity. This research is based on research led by CREA–the Community of Research on Excellence for All, and this work is funded by the European Social Fund and the Spanish Agency of Research under the Ramon y Cajal grant number RYC2018-025860-I. To this end, the current research follows a qualitative design. It implements a social media analytics strategy to identify the social discourses in social media regarding the posts shared by three newspapers around the official report on CSA in Spain. 

The current research applies social media analytics (SMA) and communicative content analysis (CCA). SMA consists of a qualitative and quantitative analysis of citizens’ debates on social media [64]. Applying a selection of keywords aligned with the topic of focus allows researchers to identify citizens’ exchanges on social media around a particular topic. In turn, CCA of SMA bases the analysis of the targeted social media interaction on a dialogic process. To this end, researchers engage in a dialogue that allows the emergence of findings that are a product of the dialogue itself [43]. Such findings would otherwise not have been unveiled in individual analyses, even after triangulation, thus contributing to their robustness [64]. 

SMA and CCA are based on the communicative methodology approach, which builds on the premise that all human beings can use language and are capable of action [65]. In this way, SMA and CCA promote a dialogic co-creation of knowledge that ensures that the voices of participants are included in the different stages of research and that these engage with researchers in a dialogue held around the scientific evidence available on the topic of focus [43]. Such an approach ensures that the research results are oriented towards the achievement of social impact [66], that is, towards the improvement of society in line with democratically set goals, such as sustainable development goals [31,34,35]. This methodology has been scientifically recognized in the EU Research Framework Programs [64].

It has proven successful at unveiling how citizens interact about topics of utmost concern in critical times [43], as well as in showing how they value and demand more scientific evidence on those issues that affect their lives [34]. Moreover, research has confirmed SMA as a valid methodology for the identification of topics of relevance to citizens on which research should focus [67].

### 2.1. Data Selection

In order to select the data, the following decisions were made:

1. Selection of the social media platform. In this research, we selected the X platform (formerly known as Twitter). This platform is commonly used by social media users to check and comment on the news online [68].
2. Selection of the time frame. On 27 October 2023, the official report by Spain’s Defensor del Pueblo (the high Commissioner of the Parliament in charge of defending citizens’ rights) on CSA in Spain was published, sparking several news publications and debates in social media. To capture these debates, selected posts published between Friday, 27 October 2023, and Sunday, 29 October 2023 (up to 13:30 h) we selected. These represented the first two and a half days after the publication of the report, in which the debate around it online was especially active.
3. Selection of the news outlets. Four newspapers that shared the news on the report were targeted for the extraction. These were “El País”, “ABC”, “Diario Feminista”, and “Periódico Educación”. The first, considered by citizens to be one of the main left-wing news outlets in Spain, was chosen due to its active engagement in promoting the focal headline. In order to provide a more nuanced picture of the debate, the second news outlet was then selected because it is considered by citizens to be one of the leading right-wing news outlets in Spain, and because it also actively shared the same headline. The third and fourth news outlets are recognized for including scientific evidence in their articles, for which they were selected. They do not identify with a political option, and their readership is considerably smaller than that of the first two. This selection accounts for the fact that online media consumption is nowadays shaped by various biases, potentially leading to audience fragmentation based on news preferences and stances on specific issues [69].
4. Selection of the keywords. In order to gather the X posts regarding the information published by the selected news outlets, the links to the news article published in each outlet were used as the search keywords. Thus, posts collected either contained any of these links, replied to a post containing any of these links, or were part of a debate in which any of the links were shared.

### 2.2. Data Extraction

Data were extracted using SMA scraper. As mentioned above, the keywords used for extraction were the links to the news articles published by the selected outlets. In sum, 325 posts were analyzed. However, 24 were deleted because they were not related to the studied topic. Table 1 illustrates the queries conducted:

The extraction of data complied with the General Data Protection Regulation (EU) 2016/679 as well as with the Terms and Conditions of X. Only those posts published by open accounts were extracted, and only information necessary for the analysis was gathered: ID, time of the post, content of the post, and link to the post. No data about the profile or the account of the platform user were retrieved. No language restrictions were applied.

### 2.3. Data Analysis

Following the abovementioned CCA, 26 researchers, of which 3 are survivors of child abuse, participated in a dialogue that allowed us to analyze and classify the identified posts in the following categories of analysis:

- Category 1. Ideological battle. This refers to the posts/debates containing arguments based on ideology. It does not refer to the orientation of the newspaper they mention but to the arguments that the citizens make in their posts around the shared news. Under this category, posts were classified as (a) ideological battle, (b) no ideological battle, or (c) not related. Within the “ideological battle” category, posts fueled a confrontation or a discussion in which citizens articulated and upheld their opinions or beliefs, markedly influenced by their individual or collective ideologies. These ideologies may have been grounded in political, religious, or cultural belief systems, or in other perspectives that determine how users interpret and address a specific issue, in this case CSA. This sometimes manifested in the form of heated debates, polarizing rhetoric, or the promotion of specific agendas that went beyond mere discussion of facts or evidence.
- Category 2. Problem-solving. This refers to the posts/debates making references to solutions to the problem of CSA. Under this category, posts were classified either as (a) yes, or (b) no.
- Category 3. Scientific evidence. This refers to the posts/debates containing scientific evidence, either in the text of the post, or in the link shared. Posts were categorized as “no scientific evidence” if they did not contain any scientific evidence. A post was considered to contain scientific evidence if (i) the post included articles from journals indexed in Scopus and/or JCR; (ii) the post claimed to be based on scientific evidence but did not reference the specific study or article; or (iii) the post included arguments that the international scientific community has already certified. Under this category, posts were classified either as (a) yes, or (b) no.
- Category 4. News outlet orientation. This refers to whether the news outlet identified with a certain ideology or was evidence-based. Under this category, posts were classified either as pertaining to a (a) mainstream news outlet or an (b) evidence-based news outlet.

The extracted posts were shared on a WhatsApp group, where the discussion took place. In case of divergence in the categorization, a consensus was reached between all participants through the established dialogue. Posts retrieved in a language other than English or Spanish were translated. Moreover, for cases in which the text was not self-explanatory, the link of the post was also analyzed. This included the analysis of images when necessary. 

In addition to the analysis of post and debate content, the engagement of each post was analyzed by looking at the number of likes, reposts, and replies.

## 3. Results

In this section, the results are divided into three distinct subsections following the analysis of the data. 

The first subsection delves into the relationship between the news outlet orientation (“Category 4” in the methodology section) and its potential role in fostering a discourse of ideological battle (defined under “Category 1”). This analysis is crucial in understanding how the perception of the media alignment with certain ideologies influences the nature of public discourse. 

The second subsection explores whether the news outlet orientation relates to a tendency for problem-solving (defined under “Category 2”) and examines whether outlets identifying with specific ideologies or those that are evidence-based are more inclined towards promoting solutions and address potential social impact or predominantly engage in ideological battles. 

Last, the third subsection aims to discern if posts grounded in scientific evidence (“Category 3”) tend to generate less debate and fewer replies but are shared more widely and receive higher approval (as indicated by a greater number of likes). This structure ensures a comprehensive analysis of the data, responding directly to our chosen criteria for scrutinizing the intricate dynamics between media orientation and public discourse within the examined dataset. 

To ensure the anonymity of individual profiles, all messages analyzed have been paraphrased in the results section.

### 3.1. Ideology-Based Media Sparks Ideological Battles

As we can observe, when purely ideological battle narratives circulate regarding abuse, either to favor certain interests or obscure others, it engendered an ideological debate devoid of any meaningful contributions to addressing the issue. The 279 posts that were built upon mainstream newspaper articles all contributed to an ideological battle. Merely sharing the news (the headline or a portion of the article) aligned with a specific ideological battle already placed it within an ideological battle context. Furthermore, these posts also gave rise to other reactions, including direct attacks on the church and expressions of outrage directed at particular groups or sectors.

As evident from the Table 2, news outlet orientation denotes whether a news source aligns with a specific ideological battle or adheres to evidence-based reporting. In this context, posts were categorized as either (a) mainstream news outlets or (b) evidence-based news outlets. Notably, the table demonstrates that no conflicts arose when information is grounded in evidence. In cases where the news is rooted in ideological battles, it encourages empty ideological debates, and, in some instances, conflicts emerge as well. All cases of ideological battles occurred around mainstream news outlets, diverging from constructive debates and potential solutions. 

As we can observe, a significant portion of the messages disseminated through X referencing certain ideological currents ignited an ideological battle on topics that deviated from productive discourse and the pursuit of solutions. Some individuals have engaged in this battle with direct criticisms, for example, targeting purely religious matters, often disregarding the victims and the potential consequences that such information could have on them. In one of the messages, the individual expresses a wish that the reality of heaven and hell turns out to be true, placing the religious individuals in the worst position imaginable (ID 143). One of the main battles was linked to the cover of the most read newspaper on the report. The cover said that the survey demonstrated that 440,000 people were abused in religious contexts. This number was obtained by multiplying the 1.13% of the sample for the population (almost 39,000,000). Periódico Educación clarified that, because the error was 1.1%, the result was between 2.23% and 0.03%, that is, that there were between more than 800,000 and less than 12,000 victims.

The 279 messages circulated through the media with the intent of fueling an ideological battle triggered responses solely rooted in ideology. These responses involved direct attacks on the government and religious institutions. For instance, one message criticizes a “study” as mere propaganda, pointing out that it is based on a telephone survey conducted by an organization lacking the authority to investigate the Church (ID 103). Additionally, these messages launch direct attacks on individuals who share accurate information about the study or its importance for victims. For example, one message calls attention to the fact that, in cases of abuse within families, there is no institution impeding investigations or detaining the victims (ID 115). It also introduces elements of debate that have no relevance to the original topic. For example, there is a mention of the study being used to divert attention from the amnesty for Puigdemont and his associates (ID 125).

A significant portion of posts partaking in ideological debates refer to criticisms against the Church as a whole. For instance, post 212 criticizes the Church for discussing abortion, the role of women in society, and morality, while being perceived as immoral. Examples include direct attacks labeling the Church as a “cult of rapists” (post 129), “fascists” (post 128), “freeloaders” (post 189), and “criminals” (post 209) or claiming that “all religions are sexist, homophobic, and supremacist” (post 242). Conversely, certain posts emerge in defense of the Church, positing that the exposure of incidents in the report aims to undermine Christianity rather than demonstrate concern for the victims (post 259). Debates extend to considerations around compensating victims and the subsequent potential fiscal implications for Spanish citizens (e.g., posts 201, 192, 228). Discussions occasionally drift to unrelated topics, such as expressing support for the King of Spain (post 169) or advocating for his removal (post 156). Additionally, some individuals label the study as “propaganda”, thereby casting doubt on its legitimacy (post 215).

The data underscore the tendency for ideological battles to emerge when discussions are rooted in mainstream news rather than evidence-based information. This can hinder the exploration of solutions and perpetuate conflict, detracting from the primary goal of constructive discourse and addressing the underlying issues at hand. 

### 3.2. Evidence-Based News Fosters Solution-Oriented Debates

As the data show, when the debate on X was grounded in scientific evidence, it became a scientific discourse that fostered solutions. The data provide compelling insight into the impact of news outlets on the nature of the discourse. In the 18 instances where evidence-based news outlets were shared, there were no discernible ideological debates that hindered the exploration of solutions or resulted from ideological biases. Notably, it was observed that in all 283 cases where mainstream news outlets were shared and failed to provide solutions to the problem, the absence of productive discourse was striking. This analysis underscores a noteworthy correlation between the promotion of solutions and evidence-based reporting. Table 3 shows the presence of solutions in the analyzed outlet orientations. 

In cases where news from evidence-based newspapers was shared, posts showed solidarity with all victims of sexual abuse, without singling out any specific sector, regardless of whether they were abused by church members or in other contexts (i.e., posts 314–316, 318–321). Additionally, there were calls for prevention and education aimed at averting such acts (i.e., 308, 310). Messages were shared stating that quality scientific teaching with values should aim to clarify available truths, distancing itself from the narratives prevalent in many media outlets (post 311).

In addition to stimulating public discourse on solutions to the issue, evidence-based media also serves the community in driving social transformations. One school has decided to incorporate an evidence-based successful educational action into its curriculum, inspired by the news and discussions on X. A direct quote from the school, following the sharing of news and various X posts, highlights the impact: “Everything I read this morning that you sent has helped shape our proposal. Even though making the Dialogic Model training open to the community and focusing on a session dedicated to participation, specifically in creating norms, was something we had in mind as we were developing the Annual General Program (AGP)”.

### 3.3. Over Double the Reposts for Evidence-Based Content

The data reveal three crucial elements: (1) the number of likes on each post, (2) the quantity of responses, and (3) the frequency of reposts. To begin, Table 4 below clearly illustrates that posts rooted in evidence-based content received almost double the number of likes in comparison to those centered on ideology. This pattern also holds true for reposts. Notably, while 57% of individuals favored posts disseminating ideological battles on the subject, a higher percentage (89%) showed appreciation for evidence-based news. Another noteworthy aspect of the analysis pertains to the responses garnered by various posts. Remarkably, posts focusing on evidence elicited no responses, as the information was presented comprehensively. This corresponds with the elevated repost rate (67%) compared to that of posts featuring ideological battle content, which, rather than sparking interest and achieving a 33% response rate, tended to incite ideological disputes, gathering a 31% response rate.

## 4. Discussion

The results of the present study are twofold. On the one hand, the analyzed posts including the media information that omitted scientific evidence of social impact triggered ideological battles on X. On the other hand, the analyzed posts that shared media based on scientific evidence of social impact garnered consensus among ideologically diverse people, encouraging actions oriented towards overcoming the issue.

The results of this study show that most of the posts related to this topic disregarded scientific evidence of social impact. Most of the arguments seen in our study revolved around ideological stances rather than scientifically proven solutions to the problem. This phenomenon contrasts with prior literature on other areas such as health. Prior evidence, such as in the case of COVID-19, has revealed that posts that contained scientific evidence were twice as likely to be reposted compared to those disseminating false information [43]. Additionally, in social networks, and especially in the health field, research has identified that false information about COVID-19 represented less than 14% in certain contexts [70].

This phenomenon is linked to the sensitivity of the topic and the tendency to engage in ideological fights concerning the issues addressed by the social sciences. The intrusion of ideology in social sciences has been widely studied in the existing literature [71,72,73,74]. In addition, in the field of sexual violence, the scarce acceptance of scientific evidence on the part of social sciences has already been highlighted by scientific literature [75]. Notably, in the case of CSA, even authors who have advocated for the decriminalization of pederasty (both in their scholarly work and/or personal lives) are often presented as references for child sexual education within the field of social sciences [76].

In the cases for which the media publications were not based on scientific evidence, the posts examined in this study frequently exhibited a tendency to avoid the data themselves when it affected individual ideologies. That is, when evidence regarding CSA negatively affected social media users within their ideological realm, these data were in most cases dismissed without any solid argument. This draws back to Copernico’s era and is exemplified in this research, for instance, when data indicating that CSA occurs across all settings of society are selectively attributed solely to a certain social group, institution, or setting. Extant research has covered the far-reaching effects across various domains of confirmation bias, wherein individuals tend to disregard information conflicting with their previous choices and judgments as a result of failing to effectively consider the strength of others’ disconfirming opinions when forming judgments [77]. 

However, when the media included scientific evidence of social impact, the X posts sharing the news consistently elicited widespread consensus and support for the presented evidence. This was exemplified in this study in all the cases where teachers or family members shared information on X and expressed their willingness to implement it as a means to prevent and overcome CSA. There is already confirmation that on the same day that the information and news from the evidence-based media were shared, a school decided to act to prevent CSA and implement dialogic model training. Furthermore, posts sharing news outlets that included scientific evidence of social impact distanced themselves from the ideology-driven posts that focused on the CSA perpetrated within certain contexts, such as the Church. On the contrary, several posts related to the evidence-based news outlets showed solidarity with all victims of CSA, regardless of who the perpetrators were or the context in which the abuse occurred. Supporting all victims is key to overcoming any form of violence and for victims to be able to become survivors [32,40]. Such social media interactions distance themselves from certain discourses that blame CSA when exercised by certain individuals or groups but hide it when exercised by “one of them”, such as by authors they consider to be references [76]. These findings suggest that a discourse on the need for scientific evidence on gender and education is being expanded on social media.

This research also confirms previous scientific literature indicating that certain media outlets prioritize gaining an audience over disseminating the truth [54]. However, the effects of media that convey scientific evidence of social impact have also generated profound social transformations aimed at overcoming gender violence in accordance with scientific research [53]. Furthermore, this type of media encourages the public to trust journalism and facilitates its gaining of social prestige [22]. 

## 5. Conclusions

The results of the present study offer valuable insights into the discussions that emerged on X regarding CSA during late October 2023, spanning a two-day period. The final sample, consisting of 279 analysis units, reveals varying patterns when it comes to both original posting and the act of sharing or reposting content. 

The SMA revealed that the ideological battle content, while sparking debates, did not receive as much engagement in the form of reposts as evidence-based information or fact-containing posts. This finding challenges the assumption that ideology-driven content is inherently more viral and suggests a complex interplay between information sharing and ideology on social media. 

In this sense, the finding that evidence-based news fostered solution-oriented debates is noteworthy. The data indicate that evidence-based news content attracts over double the number of reposts compared to other categories. This suggests that promoting evidence-based reporting in the media can potentially encourage more constructive and solution-oriented discussions, which is a crucial consideration in addressing the severe issue of CSA.

This study holds significance for the field of research on VAC, as well as other scientific fields. Whereas much research is being conducted on VAC and there is a body of literature on successful actions that contribute to preventing and overcoming it, there is still a need to deepen research on the actions that hinder or promote the implementation of such scientific evidence. This article advances knowledge in this direction. On the one hand, it shows that, among the analyzed data, social media interactions based on scientific evidence received more likes and replies than interactions based on ideology. On the other hand, it shows that when media information included scientific evidence, it contributed to changing public discourses on social issues that are often the focus of ideology battles. In particular, when the media outlets analyzed introduced scientific evidence, they promote social media interactions focused on searching for solutions or on implementing scientific evidence of social impact. Furthermore, the impact of media information based on scientific evidence does not remain in social media interactions but can have an impact in specific settings, such as schools. These findings therefore contribute relevant knowledge to the literature on how to promote the application and implementation of scientific evidence of social impact through news outlets and social media. 

These implications are not limited to the scientific evidence on how to prevent and overcome VAC but can also extend to other scientific fields, particularly within social sciences, where the discourse of scientific evidence is still hindered in many cases. Whereas in health and other life sciences, the need to promote media actions that are based on scientific evidence is clear, the same does not always apply to social sciences, as this study’s findings on ideological battles show. Indeed, this article shows that, even in issues so relevant and serious as CSA, many social media debates are ideology-driven and not focused on overcoming the problem. Such ideological debates hinder the promotion and application of scientific evidence that has already been shown to have a social impact on preventing and overcoming VAC. This study underscores the need for institutions and policies to be based on scientific evidence of social impact rather than getting in or promoting ideological battles. Future research should explore successful actions that promote evidence-based news and social media debates on social sciences-related issues.

In conclusion, these findings help us understand new approaches to content dissemination on social media. Strategies should be developed to increase the accessibility of scientific knowledge, enhance users’ critical thinking skills, and promote evidence-based reposting on social media. Understanding how different content categories behave in terms of sharing and reposting is essential for navigating the complex landscape of information dissemination in the digital age, and in doing so contributes to overcoming and preventing CSA. 

## Figures and Tables

**Table 1:** Keywords used in the data extraction.

Queries	Links
Query1–ABC	https://www.abc.es/sociedad/afirmar-espana-440000-victimas-abusos-sexuales-iglesia-20231028195425-nt.html. Accessed on 29 October 2023. https://www.abc.es/sociedad/omella-pide-perdon-victimas-abusos-afirma-cifras-20231028164004-nt.html. Accessed on 29 October 2023. https://www.abc.es/sociedad/sanchez-ensalza-informe-defensor-pueblo-sobre-abusos-20231027155552-nt.html. Accessed on 29 October 2023.
Query2–El País	https://elpais.com/sociedad/2023-10-27/presentacion-del-informe-del-defensor-del-pueblo-sobre-pederastia-en-directo.html. Accessed on 29 October 2023. https://elpais.com/sociedad/2023-10-27/la-investigacion-del-defensor-del-pueblo-estima-en-440000-las-victimas-de-pederastia-en-la-iglesia-espanola.html. Accessed on 29 October 2023. https://elpais.com/sociedad/2023-10-27/gabilondo-no-todos-los-obispos-han-colaborado-alguno-nos-ha-renido.html. Accessed on 29 October 2023. https://elpais.com/sociedad/2023-10-28/el-presidente-de-los-obispos-rechaza-el-informe-del-defensor-las-cifras-extrapoladas-por-algunos-medios-son-mentira-y-tienen-intencion-de-enganar.html. Accessed on 29 October 2023. https://elpais.com/sociedad/2023-10-29/la-dureza-del-informe-del-defensor-pilla-por-sorpresa-a-la-iglesia-y-abre-una-division-interna-sobre-como-afrontar-el-escandalo.html. Accessed on 29 October 2023.
Query3–Evidence-based journal	https://eldiariofeminista.info/2023/10/28/hay-abusos-en-todo-tipo-de-escuelas/. Accessed on 29 October 2023. https://periodicoeducacion.info/2023/10/28/docencia-de-calidad-sobre-los-abusos/. Accessed on 29 October 2023.

**Table 2:** Prevalence of ideological battles by news outlet orientation.

	Evidence-Based News Outlet	Mainstream News Outlet	Total
**Ideological battle**	0	279	279
**No ideological battle**	18	4	22
**Total**	18	283	301

**Table 3:** Presence of solutions provided according to the news outlet orientation.

Problem-Solving	Evidence-Based News Outlet	Mainstream News Outlet	Total
**Yes**	18	0	18
**No**	0	283	283
**Total**	18	283	301

**Table 4:** Post engagement according to classification as ideological battle/no ideological battle.

	Likes	Replies	Repost
	**Ideological battle**		
Post with interactions	57%	31%	33%
Post without interactions	43%	69%	67%
	**No ideological battle**		
Post with interactions	89%	0%	67%
Post without interactions	11%	100%	33%

## Data Availability

The data presented in this study are available on request from the corresponding author. The data are not publicly available due to privacy issues and anonymity and of the participants.

## References

[1] Hillis S.D., Mercy J.A., Saul J.R. (2017). The enduring impact of violence against children. Psychol. Health Med..

[2] WHO (2020). Spotlight on Adolescent Health and Well-Being. Findings from the 2017/2018 Health Behaviour in School-Aged Children (HBSC) Survey in Europe and Canada.

[3] Wang J., Iannotti R.J., Nansel T.R. (2009). School bullying among adolescents in the United States: Physical, verbal, relational, and cyber. J. Adolesc. Health.

[4] Modecki K.L., Minchin J., Harbaugh A.G., Guerra N.G., Runions K.C. (2014). Bullying prevalence across contexts: A meta-analysis measuring cyber and traditional bullying. J. Adolesc. Health.

[5] OECD (2020). Bullying. PISA 2018 Results (Volume III): What School Life Means for Students’ Lives.

[6] Andresen S. (2023). Testimonies about child sexual abuse in the family. Challenges of addressing the private sphere. Child. Abuse Negl..

[7] Seto M.C., Babchishin K.M., Pullman L.E., McPhail I.V. (2015). The puzzle of intrafamilial child sexual abuse: A meta-analysis comparing intrafamilial and extrafamilial offenders with child victims. Clin. Psychol. Rev..

[8] Kjellgren C., Carlsson C., Emilson A. (2022). Experiences with crisis management when child sexual abuse was perpetrated by staff in early childhood education: A Swedish case study. Cogent Soc. Sci..

[9] Pereira C.d.O., Pimentel R.M.M., Leitão F.N.C., Moraes S.D.T.d.A., Maia P.C.G.G.S., Santos E.V.d.L., Freitas M.N.R.d., Trigueiro G.P.D.S., Gouveia Filho P.S., Abreu L.C.d. (2020). Sexual Violence against Children and Adolescents Taking Place in Schools: An Integrative Review. Children.

[10] Pereda N., Contreras Taibo L., Segura A., Maffioletti Celedón F. (2022). An Exploratory Study on Mental Health, Social Problems and Spiritual Damage in Victims of Child Sexual Abuse by Catholic Clergy and Other Perpetrators. J. Child. Sex. Abus..

[11] Dodd K., Solomon C., Naughton M., Salmon P.M., McLean S. (2023). What enables child sexual abuse in sport? A systematic review. Trauma Violence Abus..

[12] Know Violence in Childhood (2017). Ending Violence in Childhood: Global Report 2017.

[13] Bajos N., Ancian J., Tricou J., Valendru A., Pousson J.-E., Moreau C. (2023). Child Sexual Abuse in the Roman Catholic Church in France: Prevalence and Comparison With Other Social Spheres. J. Interpers. Violence.

[14] Parent S., Lavoie F., Thibodeau M.-È., Hébert M., Blais M. (2016). Team PAJ Sexual violence experienced in the sport context by a representative sample of Quebec adolescents. J. Interpers. Violence.

[15] Alexandre J., Castro C., Gama M., Antunes P. (2022). Perceptions of sexual abuse in sport: A qualitative study in the Portuguese sports community. Front. Sports Act. Living.

[16] Bjørnseth I., Szabo A. (2018). Sexual violence against children in sports and exercise: A systematic literature review. J. Child. Sex. Abus..

[17] Langeland W., Hoogendoorn A.W., Mager D., Smit J.H., Draijer N. (2015). Childhood sexual abuse by representatives of the Roman Catholic Church: A prevalence estimate among the Dutch population. Child. Abuse Negl..

[18] Böhm B., Zollner H., Fegert J.M., Liebhardt H. (2014). Child sexual abuse in the context of the Roman Catholic Church: A review of literature from 1981–2013. J. Child Sex. Abus..

[19] Devries K.M., Child J.C., Allen E., Walakira E., Parkes J., Naker D. (2014). School violence, mental health, and educational performance in Uganda. Pediatrics.

[20] Fry D., Fang X., Elliott S., Casey T., Zheng X., Li J., Florian L., McCluskey G. (2018). The relationships between violence in childhood and educational outcomes: A global systematic review and meta-analysis. Child. Abuse Negl..

[21] Huang L., Mossige S. (2012). Academic achievement in Norwegian secondary schools: The impact of violence during childhood. Soc. Psychol. Educ..

[22] Nkuba M., Hermenau K., Goessmann K., Hecker T. (2018). Mental health problems and their association to violence and maltreatment in a nationally representative sample of Tanzanian secondary school students. Soc. Psychiatry Psychiatr. Epidemiol..

[23] Felitti V.J., Anda R.F., Nordenberg D., Williamson D.F., Spitz A.M., Edwards V., Koss M.P., Marks J.S. (1998). Relationship of childhood abuse and household dysfunction to many of the leading causes of death in adults. The Adverse Childhood Experiences (ACE) Study. Am. J. Prev. Med..

[24] Tsur N., Najjar A.A., Katz C. (2022). “Explode into small pieces”: Suicidal ideation among child sexual abuse survivors. Child. Abuse Negl..

[25] Hinduja S., Patchin J.W. (2010). Bullying, cyberbullying, and suicide. Arch. Suicide Res..

[26] Norman R.E., Byambaa M., De R., Butchart A., Scott J., Vos T. (2012). The long-term health consequences of child physical abuse, emotional abuse, and neglect: A systematic review and meta-analysis. PLoS Med..

[27] Hailes H.P., Yu R., Danese A., Fazel S. (2019). Long-term outcomes of childhood sexual abuse: An umbrella review. Lancet Psychiatry.

[28] Oliver E. (2014). Zero Violence Since Early Childhood: The Dialogic Recreation of Knowledge. Qual. Inq..

[29] Hughes K., Bellis M.A., Hardcastle K.A., Sethi D., Butchart A., Mikton C., Jones L., Dunne M.P. (2017). The effect of multiple adverse childhood experiences on health: A systematic review and meta-analysis. Lancet Public Health.

[30] Villarejo-Carballido B., Pulido C.M., de Botton L., Serradell O. (2019). Dialogic Model of Prevention and Resolution of Conflicts: Evidence of the Success of Cyberbullying Prevention in a Primary School in Catalonia. Int. J. Environ. Res. Public Health.

[31] Flecha R., Puigvert L., Racionero-Plaza S. (2023). Achieving Student Well-Being for All: Educational Contexts Free of Violence.

[32] Roca-Campos E., Duque E., Ríos O., Ramis-Salas M. (2021). The Zero Violence Brave Club: A Successful Intervention to Prevent and Address Bullying in Schools. Front. Psychiatry.

[33] Roca E., Melgar P., Gairal-Casadó R., Pulido-Rodríguez M.A. (2020). Schools that “open doors” to prevent child abuse in confinement by COVID-19. Sustain. Sci. Pract. Policy.

[34] Soler-Gallart M., Flecha R. (2022). Researchers’ Perceptions About Methodological Innovations in Research Oriented to Social Impact: Citizen Evaluation of Social Impact. Int. J. Qual. Methods.

[35] Aiello E., Donovan C., Duque E., Fabrizio S., Flecha R., Holm P., Molina S., Oliver E., Reale E. (2021). Effective strategies that enhance the social impact of social sciences and humanities research. Evid. Policy.

[36] Serradell O., Ramis M., De Botton L., Solé C. (2020). Spaces free of violence: The key role of Moroccan women in conflict prevention in schools. A case study. Indian J. Gend. Stud..

[37] Padrós M. (2014). A Transformative Approach to Prevent Peer Violence in Schools: Contributions from Communicative Research Methods. Qual. Inq..

[38] Duque E., Carbonell S., de Botton L., Roca-Campos E. (2021). Creating Learning Environments Free of Violence in Special Education Through the Dialogic Model of Prevention and Resolution of Conflicts. Front. Psychol..

[39] European Toolkit for Schools Brave’s Club: Zero Violence from Age Zero. https://www.schooleducationgateway.eu/en/pub/resources/toolkitsforschools/detail.cfm?n=5886.

[40] Rios-Gonzalez O., Ramis-Salas M. (2023). Reducing bullying in schools: Features of the zero violence brave club. Handbook of Anger, Aggression, and Violence.

[41] Rios-Gonzalez O., Puigvert Mallart L., Sanvicén Torné P., Aubert Simón A. (2019). Promoting zero violence from early childhood: A case study on the prevention of aggressive behavior in Cappont Nursery. Eur. Early Child. Educ. Res. J..

[42] Cuxart M.P., Flecha R. (2014). Towards a Conceptualization of Dialogic Leadership. IJELM.

[43] Pulido C.M., Villarejo-Carballido B., Redondo-Sama G., Gómez A. (2020). COVID-19 infodemic: More retweets for science-based information on coronavirus than for false information. Int. Sociol..

[44] Rios-Gonzalez O., Peña-Axt J.C., Legorburo-Torres G., Avgousti A., Sancho L.N. (2023). Impact of an evidence-based training for educators on bystander intervention for the prevention of violence against LGBTI+ youth. Humanit. Soc. Sci. Commun..

[45] Rodrigues de Mello R., Soler-Gallart M., Braga F.M., Natividad-Sancho L. (2021). Dialogic Feminist Gathering and the Prevention of Gender Violence in Girls with Intellectual Disabilities. Front. Psychol..

[46] Salceda M., Vidu A., Aubert A., Roca E. (2020). Dialogic Feminist Gatherings: Impact of the preventive socialization of gender-based violence on adolescent girls in out-of-home care. Soc. Sci..

[47] Melgar Alcantud P., Puigvert L., Rios O., Duque E. (2021). Language of Desire: A Methodological Contribution to Overcoming Gender Violence. Int. J. Qual. Methods.

[48] Mor N., Reich Z. (2018). From “Trust Me” to “Show Me” Journalism. Journal. Pract..

[49] Bhroin N.N., Sand S., Rasmussen T. (2021). Indigenous journalism, media innovation, and social change: A review of previous research and call for more critical approaches. Nord. Rev..

[50] Laws N., Chojnicka J. (2020). “A Future to Believe in”: Introducing Varieties of Advocacy Journalism. The Examples Sustainability and the Sanders Campaign. J. Stud..

[51] Thier K., Lin T. (2022). How Solutions Journalism Shapes Support for Collective Climate Change Adaptation. Environ. Commun..

[52] Zakharchenko A., Peráček T., Fedushko S., Syerov Y., Trach O. (2021). When Fact-Checking and “BBC Standards” Are Helpless: “Fake Newsworthy Event” Manipulation and the Reaction of the “High-Quality Media” on It. Sustain. Sci. Pract. Policy.

[53] Pulido C.M., Villarejo Carballido B., Vidu A., Ramis M., Flecha R. (2023). “Voices against Silence”: A case study of the social impact of journalism. Fem. Media Stud..

[54] Madrid A., Joanpere M., de Botton L., Campdepadrós R. (2020). Media Manipulation Against Social Justice Researchers: Second-Order Sexual Harassment. Qual. Inq..

[55] Hoewe J., Bowe B.J. (2021). Magic words or talking point? The framing of “radical Islam” in news coverage and its effects. Journalism.

[56] Ahmed S., Matthes J. (2017). Media representation of Muslims and Islam from 2000 to 2015: A meta-analysis. Int. Commun. Gaz..

[57] Chadwick A., Vaccari C., O’Loughlin B. (2018). Do tabloids poison the well of social media? Explaining democratically dysfunctional news sharing. New Media Soc..

[58] SINC El 62% de los Universitarios de España ha Vivido Situaciones de Violencia Machista. https://www.agenciasinc.es/Noticias/El-62-de-los-universitarios-de-Espana-ha-vivido-situaciones-de-violencia-machista.

[59] Francino C. (2017). SER Podcast: Escucha el La Ventana (21/02/2017—Tramo de 16:00 a 17:00) de Cadena SER.

[60] Martín-Arroyo J. (2017). La Universidad de Sevilla ignoró avisos de sus abogados sobre el catedrático abusador. El País.

[61] Freedman E., Freedman E. (2013). The Racialization of Rape and Lynching. Redefining Rape: Sexual Violence in the Era of Suffrage and Segregation.

[62] Fink K. (2019). The biggest challenge facing journalism: A lack of trust. Journalism.

[63] Lee T.-T. (2018). Virtual Theme Collection: “Trust and Credibility in News Media”. J. Mass. Commun. Q..

[64] Pulido Rodriguez C.M., Ovseiko P., Font Palomar M., Kumpulainen K., Ramis M. (2021). Capturing Emerging Realities in Citizen Engagement in Science in Social Media: A Social Media Analytics Protocol for the Allinteract Study. Int. J. Qual. Methods.

[65] Gómez A., Padrós M., Ríos O., Mara L.-C., Pukepuke T. (2019). Reaching social impact through communicative methodology. Researching with rather than on vulnerable populations: The Roma case. Front. Educ..

[66] Pulido C.M., Redondo-Sama G., Sordé-Martí T., Flecha R. (2018). Social impact in social media: A new method to evaluate the social impact of research. PLoS ONE.

[67] Cabré-Olivé J., Flecha-García R., Ionescu V., Pulido C., Sordé-Martí T. (2017). Identifying the Relevance of Research Goals through Collecting Citizens’ Voices on Social Media. Int. Multidiscip. J. Social Sci..

[68] Twitter News How Many People Come to Twitter for News? As It Turns Out, a LOT. https://blog.twitter.com/en_us/topics/insights/2022/how-many-people-come-twitter-for-news.

[69] Van der Meer T.G.L.A., Hameleers M., Kroon A.C. (2020). Crafting Our Own Biased Media Diets: The Effects of Confirmation, Source, and Negativity Bias on Selective Attendance to Online News. Mass. Commun. Soc..

[70] Massarani L., Waltz I., Leal T. (2020). COVID-19 in Brazil: An analysis about the consumption of information on social networks. J. Sci. Commun..

[71] Gunderson R. (2017). What Reproduces the Treadmill of Production?. Nat. Cult..

[72] Carvalho A. (2007). Ideological cultures and media discourses on scientific knowledge: Rereading news on climate change. Public Underst. Sci..

[73] Atienza Cerezo E., Van Dijk T.A. (2010). Identidad social e ideología en libros de texto españoles de Ciencias Sociales. Rev. Educ..

[74] Flecha R. (2022). The Dialogic Society. The Sociology Scientists and Citizens Like and Use.

[75] Yuste M., Serrano M.A., Girbés S., Arandia M. (2014). Romantic Love and Gender Violence: Clarifying Misunderstandings Through Communicative Organization of the Research. Qual. Inq..

[76] Valls R., Elboj C., Serradell O., Díez-Palomar J., Aiello E., Racionero S., Vidu A., Roca E., Joanpere M., López de Aguileta A. (2022). Promoting Admiration of Foucault Hiding his Defense of Rape and Pederasty. RIMCIS.

[77] Kappes A., Harvey A.H., Lohrenz T., Montague P.R., Sharot T. (2020). Confirmation bias in the utilization of others’ opinion strength. Nat. Neurosci..

